# Evaluation of the hypothalamic–pituitary–adrenal axis in a case series of familial partial lipodystrophy

**DOI:** 10.1186/s13098-018-0396-4

**Published:** 2019-01-03

**Authors:** Cecília Pacheco Elias, Daniela Espíndola Antunes, Michella Soares Coelho, Caroline Lourenço de Lima, Nelson Rassi, Ana Paula Meireles de Melo, Angélica Amorim Amato

**Affiliations:** 1 Unit of Endocrinology, Hospital Alberto Rassi–General Hospital of Goiânia (HGG), Avenida Anhanguera, 6479 - St. Oeste, Goiânia, GO CEP 74120-080 Brazil; 2 Unit of Endocrinology, Hospital das Clínicas, Federal University of Goiás (HC-UFG), Goiânia, Brazil; 3 Laboratory of Molecular Pharmacology, School of Health Sciences, University of Brasília (UnB), Brasília, Brazil

**Keywords:** Adipose tissue, Partial lipodystrophy, Hypothalamic–pituitary–adrenal axis, Insulin resistance, Dual X-ray energy absorptiometry

## Abstract

**Background:**

Familial partial lipodystrophy (FPL) is a rare genetic disease characterized by body fat abnormalities that lead to insulin resistance (IR). Clinical conditions linked to milder IR, such as type 2 diabetes (T2D) and metabolic syndrome, are associated with abnormalities of the hypothalamic–pituitary–adrenal (HPA) axis, but little is known about its activity in FPL.

**Methods:**

Patients meeting the clinical criteria for FPL were subjected to anthropometric, biochemical and hormone analyses. A genetic study to identify mutations in the genes encoding peroxisome proliferator-activated receptor gamma (PPARγ) was performed. Polycystic ovary syndrome and hepatic steatosis were investigated, and the patient body compositions were analyzed via dual X-ray energy absorptiometry (DXA). The HPA axis was assessed via basal [cortisol, adrenocorticotrophic hormone (ACTH), cortisol binding globulin, nocturnal salivary cortisol and urinary free cortisol (UFC)] as well as dynamic suppression tests (cortisol post 0.5 mg and post 1 mg dexamethasone).

**Results:**

Six patients (five female and one male) aged 17 to 42 years were included. In DXA analyses, the fat mass ratio between the trunk and lower limbs (FMR) was > 1.2 in all phenotypes. One patient had a confirmed mutation in the PPARγ gene: a novel heterozygous substitution of p. Arg 212 Trp (c.634C>T) at exon 5. HPA sensitivity to glucocorticoid feedback was preserved in all six patients, and a trend towards lower basal serum cortisol, serum ACTH and UFC values was observed.

**Conclusions:**

Our findings suggest that FPL is not associated with overt abnormalities in the HPA axis, despite a trend towards low-normal basal cortisol and ACTH values and lower UFC levels. These findings suggest that the extreme insulin resistance occurring in FPL may lead to a decrease in HPA axis activity without changing its sensitivity to glucocorticoid feedback, in contrast to the abnormalities in HPA axis function in T2D and common metabolic syndrome.

## Background

Familial partial lipodystrophy (FPL) is characterized by the selective loss of subcutaneous adipose tissue, such as in the limbs and gluteal area, and increased fat deposits are observed in other corporal segments, such as the face, trunk and abdomen [1–5]. Seven main FPL clinical variables have been described [1, 3–5], and, among the main genes studied, the genes encoding lamin A/C (LMNA) and peroxisome proliferator-activated receptor gamma (PPARγ) have been most thoroughly investigated [3–7]. The actual prevalence of FPL is not well established because it depends on sparse cases reported in the literature. In addition, FPL is a probable subdiagnosis of those patients who likely present with common metabolic syndrome [2, 3, 8, 9].

The loss of protective subcutaneous fat [3, 4] leads to severe insulin resistance (IR) in FPL and the early onset of related complications, such as type 2 diabetes (T2D) and other metabolic syndrome components [1–11]. The reduction of adipokines (adiponectin and leptin) may contribute to problems with insulin sensitivity [12].

Familial partial lipodystrophy carriers present phenotypic similarities with hypercortisolism and are frequently mistaken for patients with Cushing syndrome (CS) [1, 3, 6, 9]. Our hypothesis is that the dysregulation of the hypothalamic–pituitary–adrenal (HPA) axis may be involved in this syndrome, as observed in common obese states [13, 14]; however, to our knowledge, this aspect has not been specifically elucidated in FPL.

Peripheral and/or central changes in cortisol action appear to be involved in the development of metabolic syndrome, which is associated with obesity. Subtle alterations in HPA axis activity, such as elevated circulating concentrations of ACTH in the morning, changes in pulsatility [15], higher responsiveness of the HPA axis to neuropeptides [16], increased cortisol production [17] and decreased cortisol suppression in response to low doses of dexamethasone [18, 19], are observed in metabolic syndrome. The increased peripheral production of cortisol in adipose tissue caused by an increase in the expression and activity of enzyme 11β-hydroxysteroid dehydrogenase type 1 (11βHSD1) in the visceral fat deposits of obese individuals suggests a possible role for hypercortisolic tissue in metabolic syndrome [13, 20–22].

Because of the practical difficulty of molecular diagnoses, the diagnosis of FPL is essentially clinical [2, 9, 23, 24]; thus, the clinical features of this disease must be addressed. Given that lipodystrophy represents a severe phenotypic spectrum of insulin resistance, in this study, we sought to provide insights on the contribution of insulin resistance to HPA axis abnormalities.

## Methods

This paper presents a cross-sectional study that includes subjects in outpatient follow-up in the endocrinology department of two public hospitals in Goiânia, Brazil: Hospital Alberto Rassi–General Hospital of Goiânia (HGG) and Hospital das Clínicas of the Federal University of Goiás (HC-UFG). The protocol was approved by the ethics committees of these hospitals, and all patients provided written informed consent.

### Clinical and anthropometric characteristics

This study included patients with clinical characteristics of lipodystrophy subtype FPL, which is defined by the American Association of Clinical Endocrinologists (AACE) as follows: gradual loss of subcutaneous adipose tissue in extremities and/or gluteal regions with fat accumulation in intra-abdominal areas or in the face and neck; presence of acanthosis nigricans, PCOS symptoms, hypertriglyceridemia or diabetes with severe insulin resistance, evidence of hepatic steatosis, preeminent muscularity and phlebomegaly in the extremities and/or a family history of a similar phenotype of lipodystrophy [2]. The exclusion criteria were history of autoimmune diseases, patients undergoing antiretroviral therapy and patients who recently used glucocorticoids.

Anthropometric measures were obtained by the same endocrinologist for all patients, and these measurements included body weight (kg); height (m); body mass index (BMI; weight in kilograms divided by the height squared in meters); waist circumference (WC; measured from the midpoint between the costal edge and the iliac crest in cm); and hip circumference (measured at the trochanter major in cm), which was used to determine the waist-to-hip ratio (WHR). Body adiposity index (BAI) was estimated as a percentage (%) according to the following formula: BAI = (Hip circumference/Height^1,5^) − 18 [25]. The adopted metabolic syndrome criteria were those recommended by the International Diabetes Federation [26].

### Body fat evaluation

Body fat was analyzed via dual-energy X-ray absorptiometry (DXA) with the GE Lunar iDXA System (encore software version 2011, Madison, WI) to determine the following variables: total body fat (%), truncal fat (%), limb fat (%) and the android/gynoid fat ratio (A/G R). The fat mass ratio (FMR) was estimated as follows: FMR = [truncal fat (%)/[fat lower limbs (%)] [27].

### Biochemical and basal hormonal evaluation

Blood samples were collected in the morning (08:00 h) after a 12 h overnight fast. The measured biochemical variables included the glucose level, lipid profile and liver enzyme levels (enzymatic assay); glycated hemoglobin HbA1c (high-performance liquid chromatography—HPLC); insulin (chemiluminescence assay); and leptin (radioimmunoassay). The hormonal variables measured by the chemiluminescence assay included prolactin, thyrotropin (TSH), free thyroxine (T4), total testosterone, sex hormone-binding globulin (SHBG), follicle-stimulating hormone (FSH) and luteinizing hormone (LH) levels. In female patients, we also determined the dehydroepiandrosterone (DHEA), DHEA sulfate, androstenedione and 17-hydroxyprogesterone levels via radioimmunoassay.

Insulin resistance was estimated via a homeostatic model assessment (HOMA-IR) using the following formula: [insulin (mU/L) × glucose (mmol/L)/22.5].

### Ultrasonography evaluation

Hepatic steatosis was investigated via upper abdominal ultrasonography (*Philips* HD.XE, transducer 3.5 MHz). A complementary evaluation for polycystic ovary syndrome (PCOS) was performed via pelvic ultrasound using the same equipment. We considered the PCOS criteria recommended by the International Consensus of Rotterdam [28].

### Genetic studies

Venous blood samples (in EDTA collection tubes) were collected from all patients. The genetic studies were performed at the Laboratory of Molecular Pharmacology at the School of Health Sciences, University of Brasília, Brazil. Genomic DNA was extracted by a *salting out* method, and regions corresponding to exons of the PPARγ gene were amplified via PCR using specific primers. DNA sequencing was performed by the *Sanger* method.

### Evaluation of the hypothalamic–pituitary–adrenal (HPA) axis

First, a basal evaluation of the HPA axis was performed via a chemiluminescence assay, and the measured parameters included the levels of serum cortisol (08:00 h), adrenocorticotrophic hormone (ACTH), 24-h urinary free cortisol (UFC) [three samples] and nocturnal salivary cortisol (at 23:00 h). The cortisol binding globulin (CBG) levels were measured via radioimmunoassay. In a second step, the study evaluated HPA axis sensitivity to dexamethasone (DEX). Plasma cortisol was measured after low doses of oral DEX (0.5 mg and 1 mg) overnight (at 23:00 h) with a 1-week interval. HPA axis suppression was considered normal at plasma cortisol levels < 1.8 µg/dL. Patients without suppression after 1 mg DEX were administered a 2 mg DEX suppression test [29].

## Results

The study included six patients (P1–P6) who met the clinical criteria of FPL, with five females and one male (P2). The patients belonged to four unrelated families, with kinship between P1 and P2 (aunt and nephew) and between P5 and P6 (mother and daughter). The clinical, anthropometric, body composition and biochemical characteristics of the study participants are summarized in Tables 1 and 2.

**Table 1:** Clinical characteristics of patients with a clinical diagnosis of FPL syndrome

	P1	P2	P3	P4	P5	P6
Age (years)	33	20	42	17	41	24
Sex	F	M	F	F	F	F
Reason for medical consultation	Hirsutism	Family history	Hirsutism oligomenorrhea	Hirsutism	Cushing syndrome investigation	Family history
Age of TA abnormality (years)	12	8	25	Not defined	20	23
Acanthosis nigricans	+	+	+	+	+	+
Clinical lipoatrophy	Four limbs, gluteal area	Lower limbs	Four limbs, gluteal area	Slight, lower limbs	Four limbs, gluteal area	Lower limbs
Fat deposition	Face, neck	Face, neck	Face, neck, trunk	Face, neck, abdomen	Face, neck, trunk	Face, neck, trunk
Age at T2D diagnosis (years)	Glucose intolerance	–	36	–	34	–
Previous comorbidities	Dyslipidemia	–	HBP, dyslipidemia	–	HBP, dyslipidemia myocardial infarction (36 y)	–
Anti-diabetic medications	No	No	Metformin, gliclazide	No	Metformin, gliclazide	No
Other current medications	No	No	Enalapril, fibrate	No	Enalapril, HCTZ, statin, ezetimibe, fibrate	No
Family history phenotype	Brother Nephew (P2)	Father Aunt (P1)	Niece	Grandmother Aunt	Father Daughter (P6)	Grandfather Mother (P5)

*TA* adipose tissue, *T2D* type 2 diabetes, *HBP* high blood pressure, *HCTZ* hydrochlorothiazide

**Table 2:** Anthropometric, fat mass ratio and biochemical characteristics of patients with a clinical diagnosis of FPL syndrome

	P1	P2	P3	P4	P5	P6
BMI (kg/m^2^)	22.5	31.7	27.7	39.5	27.9	41.9
WC (cm)	78	96	93	122	92	110
WHR	0.92	0.92	1.02	1.07	1.0	1.02
BAI (%)	22.6	25.8	27.0	34.6	32.4	39.4
FMR	1.70	1.56	1.64	1.35	1.84	1.29
Glucose (mg/dL)	78	72	95^a^	107	142^a^	89
Insulin (mcUi/mL)	132.9	19.4	34.3	190.6	159.3	25.4
HOMA-IR	25.57	3.45	16.5	47.6	55.8	5.64
HbA1c (%)	5.8	5.8	8.9	5.8	7.6	5.5
Leptin (ng/mL)	4.1	2.8	3.7	55	17	76
ALT (U/L)	22	21	66	96	33	18
HDL (mg/dL)	26	30	21	45	22	55
Triglycerides (mg/dL)	582	163	500^b^	258	287^c^	152

*BMI* body mass index, *WC* waist circumference, *WHR* waist-to-hip ratio, *BAI* body adiposity index, *FMR* % truncal fat/lower limbs fat, *HOMA-IR* homeostatic model assessment of insulin resistance, *ALT* aspartate aminotransferase

^a^Use of the anti-diabetic medications gliclazide and metformin

^b^Use of fibrate

^c^Use of statin, fibrate and ezetimibe

Three patients had obesity defined by BMI value, and all but one had abdominal obesity. Clinical and biochemical evidence of severe insulin resistance was observed, which was disproportionate to the BMI. Moreover, a female patient (P5) was referred to the endocrinology department for an investigation of probable Cushing syndrome after a finding of precocious coronary disease (myocardial infarction at 36 years of age).

Hyperandrogenemia was detected in four females based on the elevated values of testosterone. All females met the criteria for PCOS. The male patient had normal basal levels of the gonadotropic axis.

The six patients had ultrasonographic evidence of hepatic steatosis. In the genetic study, patient P3 was found to carry the following mutation in the PPARγ gene at exon 5: a novel heterozygous substitution p.Arg212Trp (c.634C>T).

The basal and dynamic evaluations of the HPA axis are described in Table 3. We observed a tendency towards lower values of basal serum cortisol and UFC as well as circulating levels of basal ACTH, which was in the lower third of the normal range in five patients. Abnormalities in nocturnal salivary cortisol levels were not observed. All but one patient showed post-overnight 0.5 mg dexamethasone suppression of morning serum cortisol, and post-overnight 1 mg dexamethasone cortisol suppression was observed in all six participants.

**Table 3:** Evaluation of the hypothalamic–pituitary–adrenal axis in the six patients with a clinical diagnosis of FPL syndrome

Variable	P1	P2	P3	P4	P5	P6
Basal cortisol (µg/dL)	10.3	5.4	9.0	6.4	9.1	7.6
Basal ACTH (pg/mL)	16.3	9.8	12.7	8.2	5.2	17.1
CBG (µg/L)	36.6	28.3	42.4	57.4	65.0	42.2
UFC (µg/24 h)^a^	45.7 ± 10.0	36.3 ± 8.5	31.5 ± 9.0	50.4 ± 21.0	10.4 ± 4.8	31.2 ± 11.5
Salivary cortisol (nmol/L)	2.9	9.4	5.9	4.3	8.4	5.0
PDC 0.5 mg (µg/dL)	0.5	1.0	1.1	2.9	1.5	0.7
PDC 1 mg (µg/dL)	0.5	0.3	0.7	1.0	1.5	0.5

Reference values: basal cortisol: 3.7–19.4 µg/dL; ACTH 7.2–63.3 pg/mL; CBG male: 25–55/female: 40–154 µg/L; UFC: 4.3–176 µg/24 h; nocturnal salivary cortisol < 9.7 nmol/L; and CPD < 0.8 µg/dL

*ACTH* adrenocorticotrophic hormone, *CBG* cortisol binding globulin, *UFC* urinary free cortisol, *PDC* post-dexamethasone cortisol

^a^Mean ± values from three independent measurements

## Discussion

Patient clinical histories illustrate that these individuals usually seek medical attention years after the initial abnormalities of body fat distribution are detected [2, 9, 10]. Considering the challenge of the clinical diagnosis of lipodystrophy, two guidelines have already been developed in an attempt to organize clinical findings that could help with the earlier diagnosis of this disease [2, 23]. In this study, the reduction in subcutaneous adipose tissue was mild in the three youngest patients, which is consistent with a report showing that the adipose loss in FPL is gradual and progressive [2–5, 9]. The patient’s family history may represent a key parameter for the precocious identification of new and atypical cases, thus indicating the importance of screening the relatives of syndrome carriers [30].

According to the AACE consensus for lipodystrophy detection [2], PCOS represents a clinical criterion for insulin resistance, and its prevalence in FPL is approximately 25% [3]. Hyperinsulinemia results in hyperandrogenemia and anovulation [9, 31]. Therefore, the body fat distribution must be evaluated during the physical examination in females with PCOS clinical criteria.

The leptin serum concentrations among the six patients varied. Although low levels of leptin are expected during lipodystrophy, leptin assays and reference values have not been well defined for this clinical entity [2, 23]. The direct correlation between leptinemia and body fat mass appears to be well established [32]. Although BMI has already been positively correlated with leptin levels in lipodystrophy [33], this correlation is not consistently observed in patients with this disease [30, 34].

Therefore, decreases in the levels of leptin provide better therapeutic guidance than the diagnostic criteria of partial subtypes of lipodystrophy syndromes [9, 23]. Leptin replacement therapy has shown promising results in improving the metabolic outcomes of lipodystrophy syndromes, such those related to glucose metabolism, lipid profile and liver homeostasis [35].

The specific evaluation of the HPA axis in FPL represents a new aspect of the present study. This axis activity has previously been studied in antiretroviral-associated lipodystrophy, an acquired condition associated with insulin resistance related to anti-retroviral therapy. Axis hyperactivity and glucocorticoid hypersensitivity were reported in HIV-related lipodystrophy [36], despite preserved negative feedback sensitivity [37]. Our data suggest that the FPL condition may lead to a decrease in HPA axis activity, despite not changing its sensitivity to glucocorticoid feedback or cortisol circadian rhythmicity.

These findings contrast with the abnormalities in HPA axis function that were previously shown in conditions sharing phenotypic features with FPL, such as obesity, T2D and metabolic syndrome. The latter conditions have been described as pseudo-Cushing states [29] and are associated with a mild increase in UFC and an incomplete cortisol response after a 1 mg DEX test [38]. However, these signs were not observed in our study. Suppression after low oral DEX doses was also reported in other pseudo-Cushing conditions, such as obese prediabetic patients [39], although compared with healthy individuals, metabolic syndrome patients could demonstrate increased post-DEX cortisol levels, indicating a reduced ability to suppress the HPA axis [40].

The interactions between adipose tissue and the adrenal gland mainly involve obese states, and evidence suggests the possible hyperactivity of the HPA axis in obesity-related metabolic disorders [13–19, 41]. In this series of six lipodystrophy patients, however, we observed a trend of lower basal cortisol and ACTH values as well as decreased mean values for three of the analyzed UFC samples. Similar findings of low UFC have been described in female patients with abdominal obesity [42] and patients with antiretroviral-associated lipodystrophy [43]. Reduced basal cortisol concentrations were found in metabolic syndrome and attributed to an increased clearance of cortisol [14, 44].

In fact, the literature does not provide a consensus in support of a strong relationship between systemic cortisol and obesity or metabolic syndrome [45]. Most reports evaluating this linkage (adipose tissue and HPA axis) essentially consider visceral obesity. The primary disturbance in FPL is the loss of subcutaneous fat, and our findings suggest that the different types of adipocytes contribute to insulin resistance via distinct mechanisms. Although essentially speculative, these findings may also suggest that other features of the more common and milder forms of insulin resistance might indeed affect the HPA axis. Thus, it is not possible to rule out that other adipose tissue-derived factors may regulate HPA axis function. The possible stimulation of hyperinsulinemia in the HPA axis remains an undefined question [42].

Some drugs may have important implications for the interpretation of tests performed to evaluate HPA axis. However, the patients’ current medications in the present case series (described in Table 1) probably did not influence our findings. Two patients (P3 and P5) were using fenofibrate, a lipid lowering drug that can increases UFC levels assessed by HPLC, but does not affect assessment by chemiluminescence assay used in this study [29]. It is also important to note that CBG levels were normal in five of the studied patients; thus, CBG likely did not interfere with the plasma cortisol dosages and apparently did not represent an insulin resistance marker [46, 47].

Considering the phenotypic diversity among FPL patients [48], the body composition analysis by DXA constitutes an important tool for reinforcing clinical diagnoses of lipodystrophy [49]. All patients in our series had an FMR > 1.2, and the cut-off point suggested in a previous study involving FPL type 2 carriers had a sensitivity of 88.9% and a specificity of 93.8% for clinical diagnosis [50]. The index also demonstrated prognostic relevance, indicating that the severity of FPL is determined by the degree of loss of subcutaneous adipose tissue [2–4, 8, 51].

We recognize several limitations of this study. In addition to the small sample size, another limitation was the absence of a control group matched according to age and BMI. Additionally, five patients did not have a genotype diagnosis yet, representing a difficulty observed in clinical practice caused by the limited access to genetic testing. Even with advances in molecular biology, the genetic basis of the disease has not been identified for a considerable number of FPL patients [7]. We were not able to search for mutations in other candidate genes for lipodystrophy, such as LMNA, but this genetic testing represents a future project.

The HPA axis was assessed using basal measures and dynamic suppression tests to evaluate its sensitivity to dexamethasone. Since cortisol action is also regulated by at a peripheral level, we acknowledge that description of HPA axis activity would be more detailed by the evaluation of 11βHSD1 activity in adipose tissue. There is the evidence indicating increased activity of this enzyme in visceral fat in the setting of abdominal obesity and insulin resistance, thereby increasing cortisol generation from cortisone [13, 20–22, 44]. Therefore, it would be of interest to evaluate 11βHSD1 expression and activity in the visceral adipose tissue of patients with lipodystrophy syndromes.

Despite these limitations, this study highlighted important insights into a challenging disease for which early recognition is based on clinical criteria [2, 23] and broadened our knowledge of PPARγ mutations in lipodystrophy. The evaluation of HPA axis involvement in FPL provided insights into an uncertain and new aspect of the disease.

## Conclusions

In conclusion, we found no overall dysregulation of the HPA axis sensitivity to glucocorticoid feedback in patients with a clinical diagnosis of FPL and no evidence of hyperactivity. However, this topic should be further clarified for this specific condition. Our findings point to the complex effects of adipose tissue abnormalities in HPA axis function, and it is not possible to rule out that other adipose tissue-derived factors may regulate HPA axis activity.

## Abbreviations

AACE: American Association of Clinical Endocrinologists
ACTH: adrenocorticotrophic hormone
BMI: body mass index
BAI: body adiposity index
CBG: cortisol-binding globulin
CS: Cushing syndrome
DEX: dexamethasone
DXA: dual X-ray energy absorptiometry
FMR: fat mass ratio
FPL: familial partial lipodystrophy
HBP: high blood pressure
HOMA-IR: homeostatic model assessment of insulin resistance
HPA: hypothalamic–pituitary–adrenal axis
HPLC: high-performance liquid chromatography
IR: insulin resistance
PCOS: polycystic ovary syndrome
PDC: post-dexamethasone cortisol
PPARγ: peroxisome proliferator-activated receptor gamma
TA: adipose tissue
T2D: type 2 diabetes
UFC: urinary free cortisol
WC: waist circumference
WHR: waist-to-hip ratio
11βHSD1: 11β-hydroxysteroid dehydrogenase type 1
